# An Extract of *Crataegus pinnatifida* Fruit Attenuates Airway Inflammation by Modulation of Matrix Metalloproteinase-9 in Ovalbumin Induced Asthma

**DOI:** 10.1371/journal.pone.0045734

**Published:** 2012-09-21

**Authors:** In Sik Shin, Mee Young Lee, Hye Sun Lim, Hyekyung Ha, Chang Seob Seo, Jong-Choon Kim, Hyeun Kyoo Shin

**Affiliations:** 1 Basic Herbal Medicine Research Group, Korea Institute of Oriental Medicine, Daejeon, Republic of Korea; 2 College of Veterinary Medicine, Chonnam National University, Gwangju, Republic of Korea; Leiden University Medical Center, The Netherlands

## Abstract

**Background:**

*Crataegus pinnatifida* (Chinese hawthorn) has long been used as a herbal medicine in Asia and Europe. It has been used for the treatment of various cardiovascular diseases such as myocardial weakness, tachycardia, hypertension and arteriosclerosis. In this study, we investigated the anti-inflammatory effects of *Crataegus pinnatifida* ethanolic extracts (CPEE) on Th2-type cytokines, eosinophil infiltration, expression of matrix metalloproteinase (MMP)-9, and other factors, using an ovalbumin (OVA)-induced murine asthma model.

**Methods/Principal Finding:**

Airways of OVA-sensitized mice exposed to OVA challenge developed eosinophilia, mucus hypersecretion and increased cytokine levels. CPEE was applied 1 h prior to OVA challenge. Mice were administered CPEE orally at doses of 100 and 200 mg/kg once daily on days 18–23. Bronchoalveolar lavage fluid (BALF) was collected 48 h after the final OVA challenge. Levels of interleukin (IL)-4 and IL-5 in BALF were measured using enzyme-linked immunosorbent (ELISA) assays. Lung tissue sections 4 µm in thickness were stained with Mayer’s hematoxylin and eosin for assessment of cell infiltration and mucus production with PAS staining, in conjunction with ELISA, and Western blot analyses for the expression of MMP-9, intercellular adhesion molecule (ICAM)-1 and vascular cell adhesion molecule (VCAM)-1 protein expression. CPEE significantly decreased the Th2 cytokines including IL-4 and IL-5 levels, reduced the number of inflammatory cells in BALF and airway hyperresponsiveness, suppressed the infiltration of eosinophil-rich inflammatory cells and mucus hypersecretion and reduced the expression of ICAM-1, VCAM-1 and MMP-9 and the activity of MMP-9 in lung tissue of OVA-challenged mice.

**Conclusions:**

These results showed that CPEE can protect against allergic airway inflammation and can act as an MMP-9 modulator to induce a reduction in ICAM-1 and VCAM-1 expression. In conclusion, we strongly suggest the feasibility of CPEE as a therapeutic drug for allergic asthma.

## Introduction

Allergic asthma is a common pulmonary disease of which the prevalence has increased substantially in recent decades [1]. It is a chronic inflammatory disease that is characterized by hyperresponsiveness and inflammation of the airway. This inflammation is associated with the infiltration of eosinophils, T helper type 2 (Th2) lymphocytes and neutrophils from blood to airway and to lung tissue [2]. The infiltrating inflammatory cells contribute to the production of Th2 cytokines and chemokines, which can cause airway hyperresponsiveness [3].

Inflammatory cells infiltration is regarded as a critical step in acute and chronic inflammation in lung tissue. Recruitment of leukocytes from the circulating blood into tissues is controlled by sequential activation of adhesion molecules and their ligands on leukocytes, epithelial cells and vascular endothelial cells [4], [5]. It was recently reported that inflammatory cell infiltration is partly caused by enhanced adhesion of leukocytes to epithelial cells via the expression of adhesion molecules [6]. Up-regulation of adhesion molecules such as intercellular adhesion molecule (ICAM)-1 and vascular cell adhesion molecule (VCAM)-1 on lung airway epithelium is associated with inflammatory cell infiltration, critical for the pathogenesis of various airway inflammatory diseases [7].

**Figure 1:**
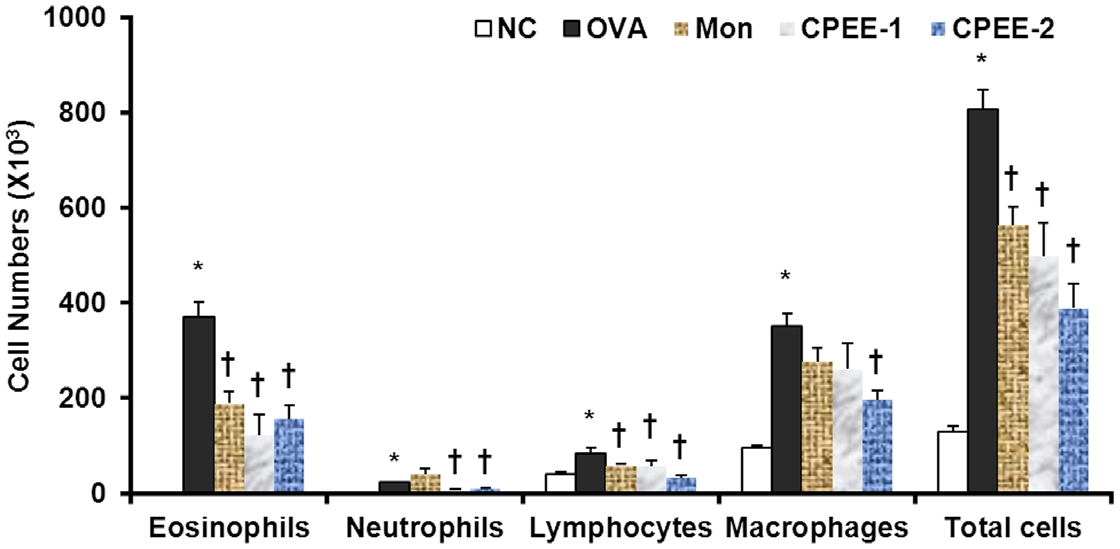
CPEE inhibits the recruitment of inflammatory cells in bronchoalveolar lavage fluid (BALF) of mice. Cells were isolated by centrifugation and stained with Diff-Quik® stain reagent. Cell numbers were determined using a light microscope to count cells in at least five squares of a hemocytometer after excluding dead cells using Trypan blue. NC; normal control mice treated with PBS only; OVA; OVA-sensitized/challenged mice; Mon; Montelukast (30 mg/kg) + OVA-sensitized/challenged mice; CPEE-1; CPEE (100 mg/kg) + OVA-sensitized/challenged mice; CPEE-2; CPEE (200 mg/kg) + OVA-sensitized/challenged mice. Values are expressed as mean ± SD (n = 6/group). *Significantly different from NC, *P*<0.05; †significantly different from OVA, *P*<0.05.

The matrix metalloproteinases (MMPs) are a family of over 20 different proteases with varying abilities to cleave components of the extracellular matrix (ECM). The MMPs play an important role in the tissue remodeling associated with various pathological processes such as morphogenesis, angiogenesis, tissue repair, migration and metastasis [8]. MMP-9 in particular appears to play a role in chronic airway inflammation and remodeling in asthma, and in a murine asthma model is upregulated in association with the accumulation of inflammatory cells [9], [10]. Moreover, recent studies in a murine allergy model have revealed that MMP inhibitors regulate inflammatory cell infiltration though a reduction in ICAM-1 and VCAM-1 expression [7]. These findings suggest the feasibility of MMP-9 as a therapeutic target in lung inflammatory diseases.

**Figure 2:**
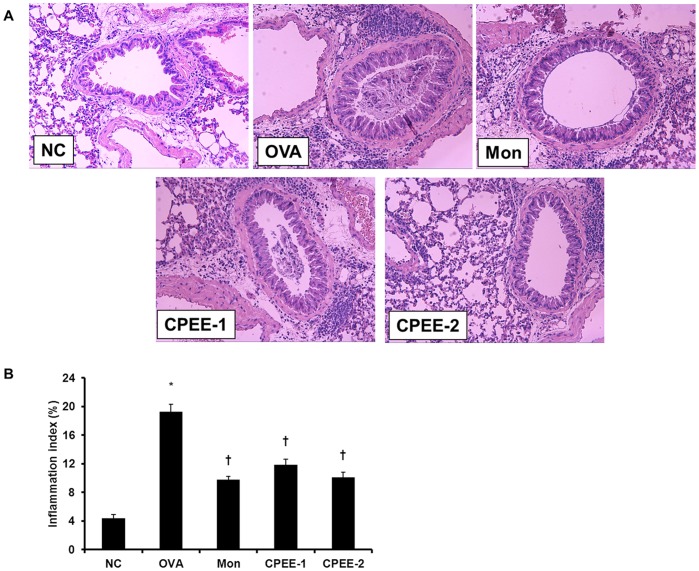
CPEE inhibits the recruitment of inflammatory cells to lung tissue of mice. (A) Histological examination of lung tissue was performed 48 h after the last OVA challenge. Lung tissues were fixed, sectioned at 4 µm thickness, and stained with H&E solution (magnification x200). (B) Scoring the extent of inflammation by quantitative analysis of inflammatory cell infiltration in lung sections were performed using an image analyzer (Molecular Devices Inc., CA, USA). Quantitative analysis was assessed in at least four squares of a sample slide stained with H&E. NC; normal control mice treated PBS only; OVA; OVA-sensitized/challenged mice; Mon; Montelukast (30 mg/kg) + OVA-sensitized/challenged mice; CPEE-1; CPEE (100 mg/kg) + OVA-sensitized/challenged mice; CPEE-2; CPEE (200 mg/kg) + OVA-sensitized/challenged mice. Values are expressed as mean ± SD (n = 6/group). *Significantly different from NC, *P*<0.05; †significantly different from OVA, *P*<0.05.

*Crataegus pinnatifida* (Chinese hawthorn) has long been used as a herbal medicine in Asia and Europe. It has been used for the treatment of various cardiovascular diseases such as myocardial weakness, tachycardia, hypertension and arteriosclerosis. Recent studies were reported that *Crataegus pinatifida* ethanolic extracts (CPEE) have beneficial effects, including antioxidant and anti-inflammatory effects and protective effects in cardiovascular diseases. Kao et al. [11] reported that CPEE decreased the level of prostaglandin E2 and nitric oxide induced in macrophage RAW 264.7 cells by lipopolysaccharide. In addition, CP possesses free-radical scavenging activity and reduces the paw edema in a carrageenam-induce paw edema model [12]. These effects of CPEE have been associated with various active ingredients of CPEE, which a previous study has reported to be vitexin-2-*O*-rhamnoside, rutin and hyposide [13]. The anti-oxidant and anti-inflammatory effects of these active ingredients were also demonstrated in various in vivo and in vitro experiments [14]–[16].

**Figure 3:**
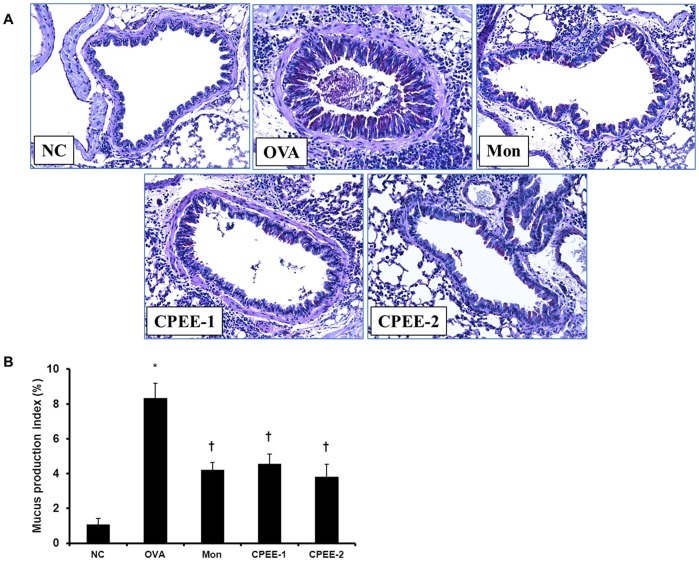
CPEE reduces mucus production in lung tissues of mice. (A) Histological examination of mucus secretion in lung tissue 48 h after the last OVA challenge. Lung tissue was fixed, sectioned at 4 µm thickness, and stained with periodic acid Schiff (PAS) for mucus production (magnification ×200). (B) Scoring of mucus production in lung sections were performed an image analyzer (Molecular Devices Inc., CA, USA). Quantitative analysis was assessed in at least four squares of a sample slide stained with PAS. NC; normal control mice treated with PBS only; OVA; OVA-sensitized/challenged mice; Mon; Montelukast (30 mg/kg) + OVA-sensitized/challenged mice; CPEE-1; CPEE (100 mg/kg) + OVA-sensitized/challenged mice; CPEE-2; CPEE (200 mg/kg) + OVA-sensitized/challenged mice. Values are expressed as mean ± SD (n = 6/group). *Significantly different from NC, *P*<0.05; †significantly different from OVA, *P*<0.05.

Based on the effects of CPEE and its active ingredients, we proposed that CP may reduce airway inflammation in allergic asthma. Therefore, we examined the anti-inflammatory effect of CP on Th2-type cytokines, eosinophil infiltration and other factors using an OVA-induced murine asthma model, and attempted to determine the possible role of MMP-9 in the protective effects of CP. In addition, we investigated the effects of CPEE on the expression of ICAM-1, VCAM-1, and MMP-9.

## Materials and Methods

### Preparation of CPEE

The dried fructus of Crataegus pinnatifida (200 g) were extracted three times by sonication for 1 h with 2 L 70% ethanol. The extracted solution was filtered through filter paper and evaporated to dryness (70.9 g). The yield of ethanolic extract obtained was 35.5%.

### Animals and Experimental Procedure

Specific pathogen-free female BALB/c mice (7 weeks old) were purchased from the Orient Co. (Seoul, Korea) and used after a week of quarantine and acclimatization. The mice were allowed sterilized tap water and standard rodent chow. All experimental procedures were carried out in accordance with the NIH Guidelines for the Care and Use of Laboratory Animals and were approved by Korea Institute of Oriental Medicine Institutional Animal Care and Use Committee. The animals were cared for in accordance with the dictates of the National Animal Welfare Law of Korea.

**Figure 4:**
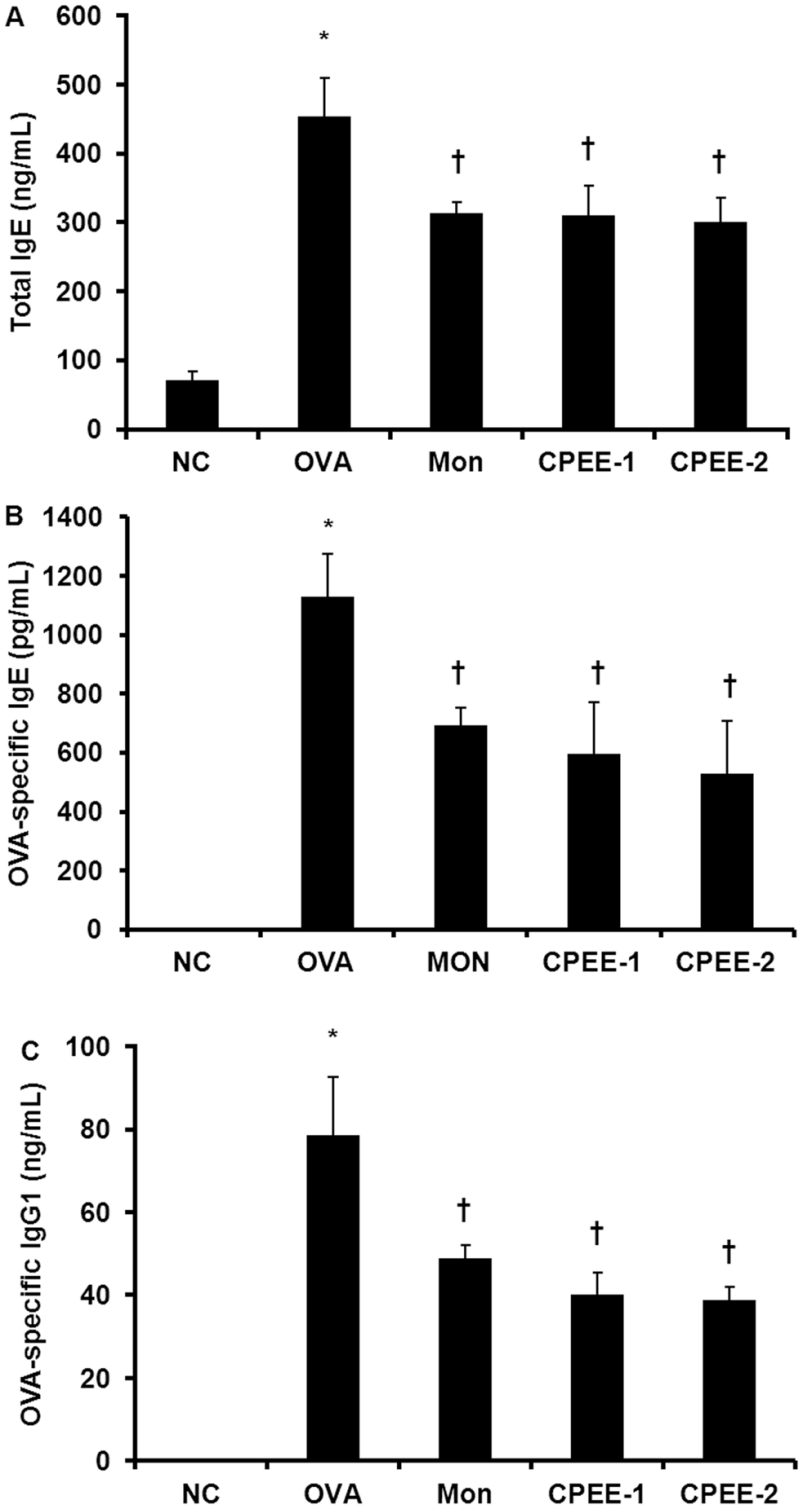
CPEE reduces total IgE, OVA-specific IgE, and OVA-specific IgG1 levels in serum of mice. (A) Total IgE level (B) OVA-specific IgE level (C) OVA-specific IgG1 level. NC; normal control mice treated with PBS only; OVA; OVA-sensitized/challenged mice; Mon; Montelukast (30 mg/kg) + OVA-sensitized/challenged mice; CPEE-1; CPEE (100 mg/kg) + OVA-sensitized/challenged mice; CPEE-2; CPEE (200 mg/kg) + OVA-sensitized/challenged mice. Values are expressed as mean ± SD (n = 6/group). *Significantly different from NC, *P*<0.05; †significantly different from OVA, *P*<0.05.

OVA sensitization and airway challenge were performed as previously described [17]. In brief, mice were sensitized on day 0 and 14 by intraperitoneal injection of 20 µg OVA emulsified in 2 mg aluminum hydroxide in 200 µL PBS buffer (pH 7.4). On days 21, 22 and 23 after initial sensitization, mice received an airway challenge with OVA (1%, w/v, in PBS) for 1 h using an ultrasonic nebulizer (NE-U12; Omron Corp., Tokyo, Japan). CPEE was dissolved in PBS and was freshly prepared daily before treatment. CPEE was administered by gavage to mice at doses of 100 mg/kg or 200 mg/kg once daily from day 18 to 23. Some previous studies used 250 mg/kg or 500 mg/kg as oral doses [18], [19]. In preliminary studies, we used the dose levels of 200 mg/kg and 400 mg/kg as effective doses. The procedure of preliminary studies is consistent with those of this study. In BALF, inflammatory cell counts were decreased in 200 mg/kg and 400 mg/kg of CPEE treated mice compared with OVA-challenged mice. However, 200 mg/kg treated mice more decreased inflammatory cell counts than 400 mg/kg treated mice. Therefore, based on the preliminary studies, we used 100 mg/kg and 200 mg/kg of CPEE as oral doses in this study. Negative and positive control mice were orally administered PBS or montelukast (30 mg/kg in PBS), respectively. Montelukast was developed as a cysteinyl leukotriene (cys-LT)-1 receptor antagonist [20]: after the clinical responses in patients with aspirin-sensitive asthma, nocturnal exacerbations of asthma and allergic asthma, montelukast was successfully introduced into the market [21].

**Figure 5:**
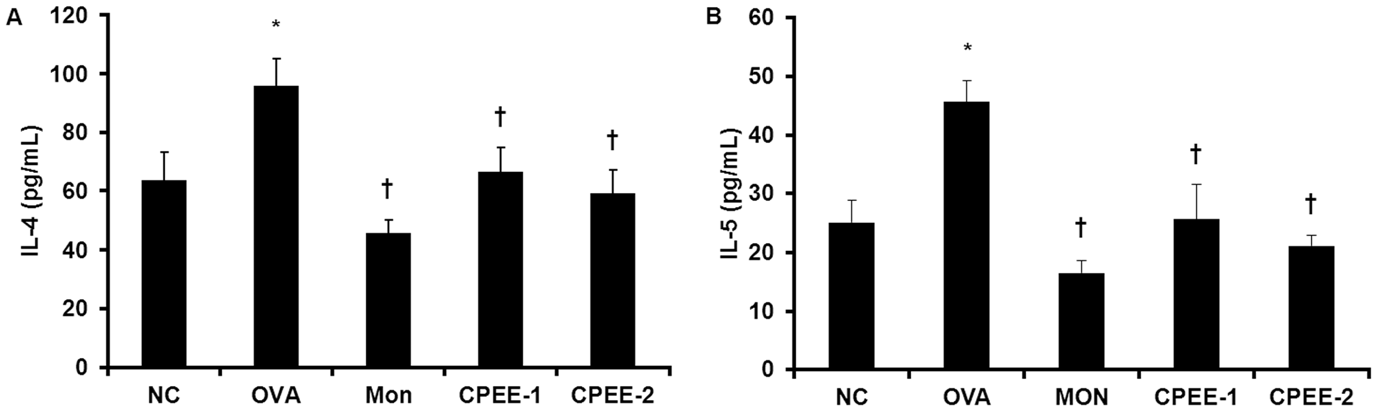
CPEE reduces the levels of IL-4 and IL-5 in BALF of mice. (A) IL-4 level (B) IL-5 level. NC; normal control mice treated with PBS only; OVA; OVA-sensitized/challenged mice; Mon; Montelukast (30 mg/kg) + OVA-sensitized/challenged mice; CPEE-1; CPEE (100 mg/kg) + OVA-sensitized/challenged mice; CPEE-2; CPEE (200 mg/kg) + OVA-sensitized/challenged mice. Values are expressed as mean ± SD (n = 6/group). *Significantly different from NC, *P*<0.05; †significantly different from OVA, *P*<0.05.

Additional experiment was performed as first study to investigate action mechanism of CPEE. Experimental groups were divided into six including NC (normal control group), OVA (OVA-sensitization/challenge), Mon (OVA-sensitization/challenge + montelukast (30 mg/kg)), CPEE-2 (OVA-sensitization/challenge + CPEE (200 mg/kg)), CPEE-2+ MMPI (OVA-sensitization/challenge + CPEE (200 mg/kg) + MMPI-1 (20 mg/kg), MMPI (OVA-sensitization/challenge + MMPI-1 (20 mg/kg). CPEE and montelukast was administered by oral gavage once daily from day 18 to 23. MMP-9 inhibitor I (MMPI-I;IC50 for MMP-9 = 150 mol/L; 4-Abz-Gly-Pro-D-Leu-D-Ala-NHOH; Calbiochem, San Diego, Calif) was used to inhibit MMP-9 activity. MMPI-I (20 mg/kg, i.p.) dissolved in distilled water was administered 3 times of 24 hour intervals, beginning 30 minutes before the OVA challenge.

**Figure 6:**
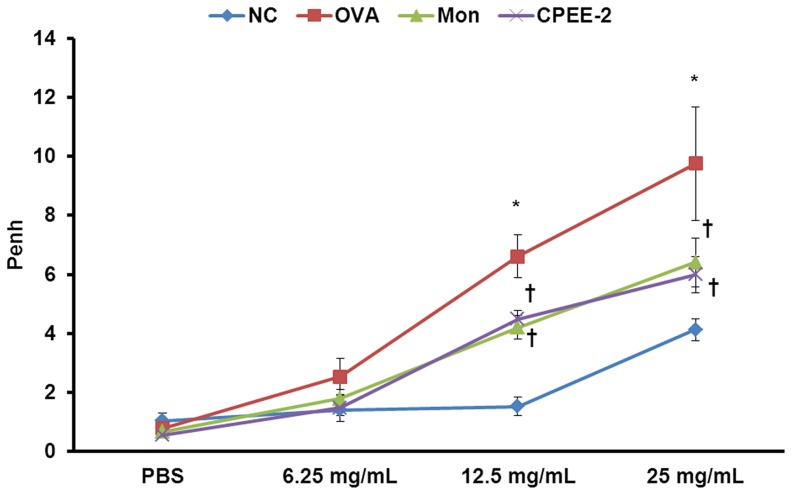
CPEE reduces airway hyperresponsiveness (AHR) by OVA-challenge. AHR was measured using plethysmography 24 h after the final OVA challenge, in mice given various doses of methacholine (6.25–25 mg/mL). NC; normal control mice treated with PBS only; OVA; OVA-sensitized/challenged mice; Mon; Montelukast (30 mg/kg) + OVA-sensitized/challenged mice; CPEE-2; CPEE (200 mg/kg) + OVA-sensitized/challenged mice. Values are expressed as mean ± SD (n = 6/group). *Significantly different from NC, *P*<0.05; †significantly different from OVA, *P*<0.05.

### Inflammatory Cell Counts in Bronchoalveolar Lavage Fluid (BALF)

Following OVA challenge of mice, BALF samples were obtained and processed, and inflammatory cells were counted as previously described [17]. In brief, mice were sacrificed by intraperitoneal injection of pentobarbital (50 mg/kg, Hanlim Pharm. Co., Seoul, Korea) 48 h after the last challenge, and a tracheostomy was performed. To obtain BALF, ice-cold PBS (0.6 mL) was infused into the lung and withdrawn via tracheal cannulation three times (total volume 1.8 mL). Total inflammatory cell numbers were assessed by counting cells in at least five squares of a hemocytometer after exclusion of dead cells by Trypan blue staining. To determine differential cell counts, 100 µL of BALF was centrifuged onto slides using a Cytospin (Hanil Science Industrial, Seoul, Korea) (200 g, 4°C, 10 min). After slides were dried, cells were fixed and stained using Diff-Quik® staining reagent (B4132-1A; IMEB Inc., Deerfield, IL), according to the manufacturer’s instructions. The supernatant of the BALF was stored at −70°C for cytokine measurements.

**Figure 7:**
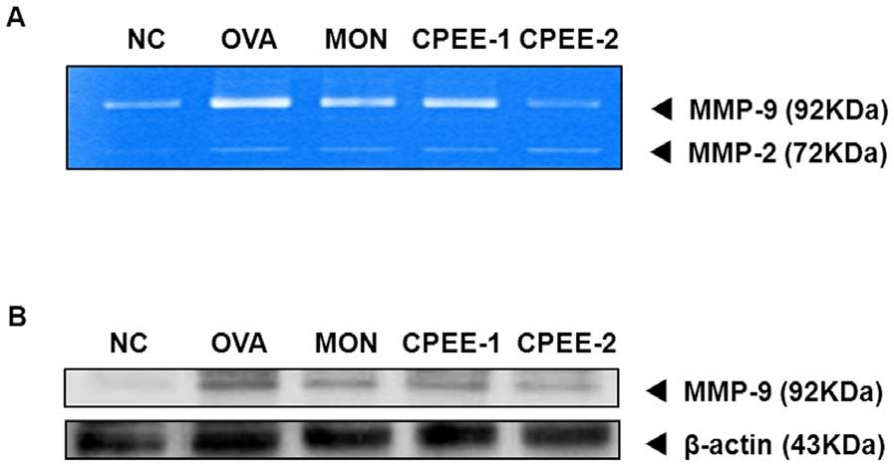
CPEE decreases the MMP-9 activities and protein expression in lung tissues of mice. The protein of supernatants was loaded for gelatin zymography (60 µg/lane). SDS−PAGE zymography was performed according to the method of Heussen and Dowdle (1980). (A) MMP-9 activities (B) MMP-9 protein expression. NC; normal control mice treated with PBS only; OVA; OVA-sensitized/challenged mice; Mon; Montelukast (30 mg/kg) + OVA-sensitized/challenged mice; CPEE-1; CPEE (100 mg/kg) + OVA-sensitized/challenged mice; CPEE-2; CPEE (200 mg/kg) + OVA-sensitized/challenged mice.

### Measurements of Total IgE, OVA-specific IgE, and OVA-specific IgG1 in Serum

Total IgE, OVA-specific IgE and OVA-specific IgG1 were measured by enzyme-linked immunosorbent assay (ELISA). Briefly, 96-well microtiter plates were coated overnignt with isotype-specific coating (total IgE) and 10 µg/mL OVA in PBS-Tween 20 (OVA-specific IgE and OVA-specific IgG1). After washing and blocking of plate, samples were incubated for 2 hours. Subsequently, 96-well plates were washed, and HRP-conjugated goat anti-mouse IgE (total IgE and OVA specific IgE) and anti-mouse IgG1 antibody (OVA-specific IgG1) were added. After washing four times, 200 µL of o-phenylenediamine dihydrochloride (Sigma-Aldrich, St. Louis, MO) was added to each well. The plate was incubated for 10 min in the dark and then absorbance was determined at 450 nm using a microplate ELISA reader (Bio-Rad Laboratories, CA, USA). Total IgE, OVA-specific IgE, and OVA-specific IgG1 concentrations were calculated from a standard curve generating using 250 ng/mL recombinant IgE and IgG1 (Serotec, Oxford, UK). These experiments were performed three times.

**Figure 8:**
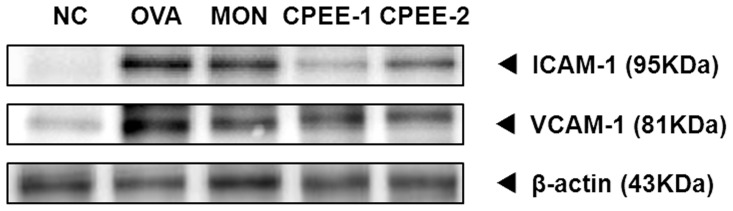
CPEE reduces the expression of ICAM-1 and VCAM-1 proteins in lung tissues of mice. NC; normal control mice treated with PBS only; OVA; OVA-sensitized/challenged mice; Mon; Montelukast (30 mg/kg) + OVA-sensitized/challenged mice; CPEE-1; CPEE (100 mg/kg) + OVA-sensitized/challenged mice; CPEE-2; CPEE (200 mg/kg) + OVA-sensitized/challenged mice. Values are expressed as mean ± SD (n = 6/group). *Significantly different from NC, *P*<0.05; †significantly different from OVA, *P*<0.05.

### Measurements of IL-4, IL-5, IL-13 and Eotaxin in BALF

Levels of IL-4, IL-5, IL-13 and eotaxin in BALF were quantified by ELISA (BioSource International, Camarillo, CA) according to the manufacturer’s protocols. These experiments were performed three times.

**Table 1:** CPEE reduces the level of Th2 cytokines and eotaxin in BALF.

Groups	IL-4 (pg/mL)	IL-5 (pg/mL)	IL-13 (pg/mL)	Eotaxin (pg/mL)
**NC**	49.5±19.3	51.9±10.2	16.4±5.9	42.1±13.4
**OVA**	105.4±13.5*	93.1±11.3*	39.5±5.8*	71.3±11.2*
**Mon**	72.2±12.4†	55.7±9.3†	23.6±5.7†	43.4±8.9†
**CPEE-2**	71.0±10.2†	66.8±6.4†	29.2±4.8†	43.6±85†
**CPEE-2+MMPI-I**	58.2±12.6†	56.2±11.3†	27.7±4.1†	35.8±9.9†
**MMPI-I**	79.7±17.3†	66.2±10.6†	34.3±4.0	46.6±4.2†

NC; normal control mice treated with PBS only; OVA; OVA-sensitized/challenged mice; Mon; Montelukast (30 mg/kg) + OVA-sensitized/challenged mice; CPEE-2; CPEE (200 mg/kg) + OVA-sensitized/challenged mice; CPEE-2+ MMPI-I; CPEE (200 mg/kg) + MMPI-I (20 mg/kg) + OVA-sensitized/challenged mice; MMPI-I; MMPI-I (20 mg/kg).

Values are expressed as mean ± SD (n = 7/group).

* Significantly different from NC, *P*<0.05;

† significantly different from OVA, *P*<0.05.

### Measurement of Airway Hyperresponsiveness (AHR)

Twenty-four hours after the final aerosol challenge, AHR was assessed in conscious and unrestrained mice by means of whole-body plethysmography (OCP3000 instrument; Allmedicus, Seoul, Korea). Each mouse was plasced in a plastic chamber and exposed to aerosolized PBS, for 3 min at each exposure level. Bronchoconstriction was recored for an additional 5 min after each methacholine dose. The highest Penh value obtained during each methacholine challenge was expressed as a proportion of the basal Pehn value seen in response to PBS challenge.

**Figure 9:**
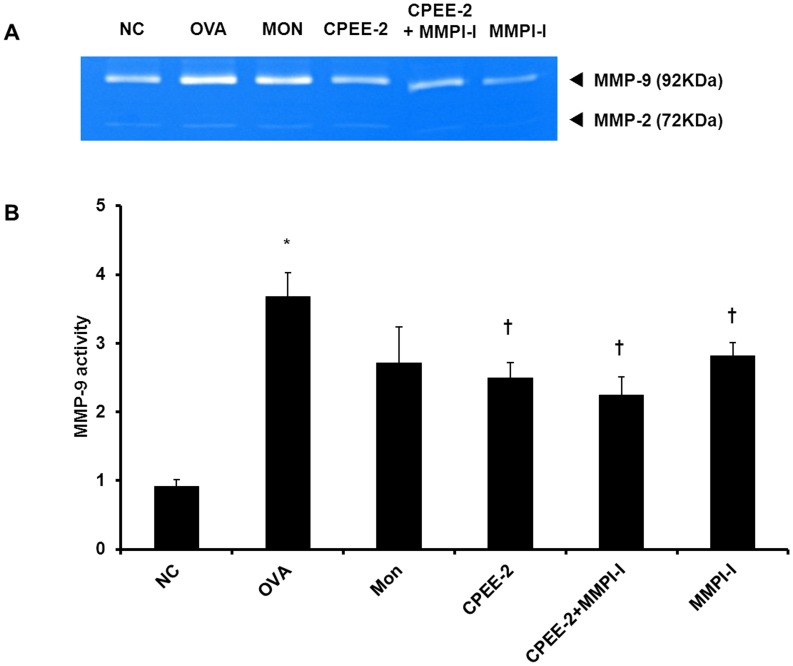
CPEE reduces the activity of MMP-9. The protein of supernatants was loaded for gelatin zymography (60 µg/lane). SDS−PAGE zymography was performed according to the method of Heussen and Dowdle (1980). (A) MMP-9 activities, (B) Quantitative analysis of MMP-9. NC; normal control mice treated with PBS only; OVA; OVA-sensitized/challenged mice; Mon; Montelukast (30 mg/kg) + OVA-sensitized/challenged mice; CPEE-2; CPEE (200 mg/kg) + OVA-sensitized/challenged mice; CPEE-2+ MMPI-I; CPEE (200 mg/kg) + MMPI-I (20 mg/kg) + OVA-sensitized/challenged mice; MMPI-I; MMPI-I (20 mg/kg) + OVA-sensitized/challenged mice. Values are expressed as mean ± SD (n = 6/group). *Significantly different from NC, *P*<0.05; †significantly different from OVA, *P*<0.05.

### Measurement of MMP-9 in Lung Tissue

Lung tissues were homogenized (1/10 w/v) in tissue lysis/extraction Extraction reagent plus protease inhibitor (Sigma-Aldrich) to obtain extracts of lung tissues. After centrifugation (12,000 g, 4°C, 10 min), the protein concentration in the supernatants was determined using a protein assay reagent (Bio-Rad Laboratories) according to the manufacturer’s instructions, and equal amounts of total protein were loaded for gelatin zymography (60 µg/lane). Sodium dodecyl sulfate−polyacrylamide gel electrophoresis (SDS−PAGE) zymography to determine the gelatinase activity was performed according to Heussen and Dowdle [22]. Briefly, zymogram gels consisted of 10% SDS-PAGE containing 1% gelatin as an MMP substrate. The gels were washed in 2.5% Triton X-100 for 1 h to remove SDS, then incubated at 37°C for 16 h in developing buffer (1M Tris-HCl, pH 7.5 with CaCl_2_). Thereafter gels were stained with 25% methanol/8% acetic acid containing Coomassie brilliant blue. Gelatinase activity was visualized as white bands on a blue background, representing areas of proteolysis of the substrate protein. These experiments were performed three times.

**Figure 10:**
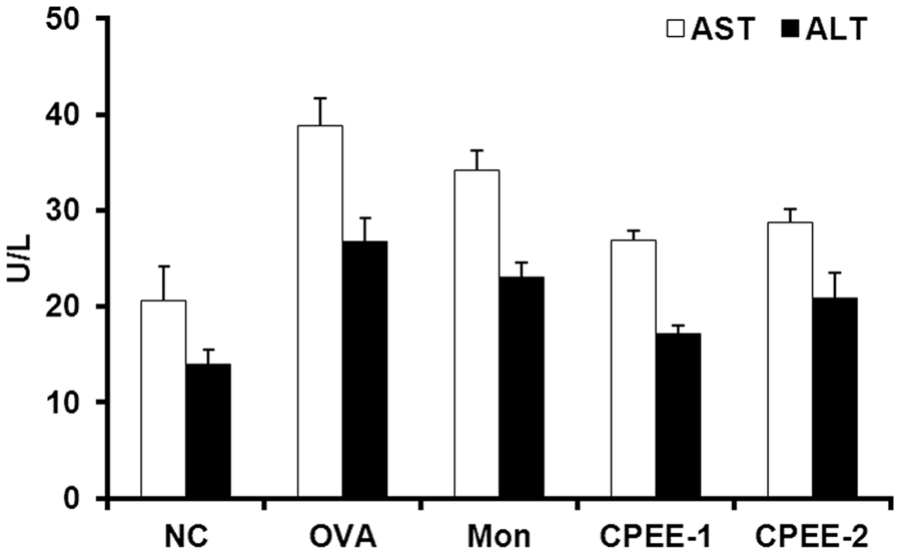
CPEE has no effect on AST and ALT levels in serum of mice. Each sample was analyzed using commercial kits (Beckman Coulter, Inc., Fullerton, CA).

### Western Blot

Equal amounts of total lung protein (30 µg) were heated at 100°C for 5 min then loaded on 8% SDS−PAGE gels, followed by transfer to nitrocellulose membrane (at 100 V for 2 h). The membranes were blocked for 1 h with Tris-buffered saline containing 0.05% Tween-20 (TBST) plus 5% skim milk and were incubated with an anti-ICAM-1 (1∶1000 dilution; Abcam, Cambridge, UK), anti-VCAM-1 (1∶1000 dilution; Abcam), anti-MMP-9 (1∶1000 dilution; Santa Cruz Biotechnology, Santa Cruz, CA) and anti-β-actin (1∶1000 dilution; Cell Signaling Technology, Danvers, MA) overnight at 4°C. The membranes were washed three times with TBST then incubated with a 1∶10000 dilution of horseradish peroxidase (HRP)-conjugated secondary antibody (Jackson ImmunoResearch, West Grove, PA) for 1 h at room temperature. The membranes were again washed three times with TBST then developed using an enhanced chemiluminescence kit (ECL, Amersham, UK). These experiments were performed three times.

### Histology

After BALF samples were obtained, lung tissue was fixed in 10% (v/v) neutral buffered formalin. Tissues were embedded in paraffin, sectioned at 4 µm thickness, and stained with H&E solution (hematoxylin, Sigma MHS-16, and eosin, Sigma HT110-1-32) and periodic acid−Schiff (PAS) (IMEB Inc., San Marcos, CA) to estimate inflammation and mucus production, respectively. Quantitative analysis of inflammation and mucus production was performed using an image analyzer (Molecular Devices Inc., CA, USA). Quantitative analysis was assessed in at least four squares of a sample slide stained with H&E and PAS.

### Measurement of Alanine Aminotransferase (ALT) and Aspartate Aminotransferase (AST) Levels in Serum

ALT and AST levels to assess liver function were determined using an autoanalyzer (Beckman CX4; Beckman Coulter Inc., Fullerton, CA) and commercial kits (Beckman Coulter, Inc.). These experiments were performed three times.

### Image Capture and Photomicrography

Photomicrographs were obtained using a Photometric Quantix digital camera running a Windows program, and montages were assembled in Adobe Photoshop 7.0. Images were cropped and corrected for brightness and contrast, but were not otherwise manipulated.

### Statistical Analysis

Data are expressed as means ± standard deviations. Statistical significance was determined using the analysis of variance (ANOVA) followed by a multiple comparison test with a Bonferroni adjustment. *P* values of <0.05 were considered significant.

## Results

### CPEE Reduces the Number of Eosinophils and other Inflammatory Cells in BALF of OVA-Challenged Mice

To evaluate the anti-inflammatory effects of CPEE, total cell numbers and eosinophil, macrophage and neutrophil counts in BALF were measured. As shown in Figure 1, OVA-challenged mice showed significant increases in the numbers of eosinophil and other inflammatory cells compared with the negative control mice. In contrast, CPEE-treated OVA-challenged mice showed a significantly decreased influx of eosinophils and macrophages into the airways compared with the control OVA-challenged mice.

### CPEE Decreases Inflammatory Cell Infiltration and Mucus Production in Lung Tissue of OVA-challenged Mice

We observed a marked infiltration of inflammatory cells into the peribronchiole and perivascular connective tissue in lung tissues section from OVA-challenged mice (Fig. 2). Most infiltrated infiltrating cells were eosinophils. However, CPEE significantly decreased this eosinophil-rich inflammatory cell infiltration compared with control OVA-challenged mice.

In lung sections stained with PAS, overproduction of mucus and goblet cell hyperplasia were observed in bronchial airways of OVA-challenged mice (Fig. 3). In contrast, CPEE-treated OVA-challenged mice showed a reduction in the number of PAS-stained goblet cells.

### CPEE Reduces the Levels of Total IgE, OVA-specific IgE, and OVA-specific IgG1a in Serum of OVA-challenged Mice

Total IgE, OVA-specific IgE, and OVA-specific IgG1 levels for each experimental group were determined by ELISA 48 h after the final OVA challenge. OVA-challenged mice demonstrated dramatically elevated levels of IgE and IgG1 in serum compared with those seen in the negative controls. When compared with the OVA-challenged group, mice that also received CPEE exhibited reduced total IgE, OVA-specific IgE, and OVA-specific IgG1 levels in serum (Fig. 4).

### CPEE Reduces the Release of IL-4 and IL-5 in BALF of OVA-challenged Mice

As shown in Figure 5, the levels of IL-4 and IL-5 were significantly increased in OVA-challenged mice compared with negative controls. Administration of CPEE induced a significant decrease in these increased levels of IL-4 and IL-5 in BALF of OVA-challenged mice.

### CPEE Reduces Airway Hyperresponsiveness of OVA-challenged Mice

The Pehn value of the OVA-challenged mice was significantly higher than that of the PBS control group at any concentration between 6.25 and 25 mg/mL of methacholine (Figure 6). In the CPEE treated mice, the Penh value was significantly reduced compared to that of the OVA-challenged mice. Montelukast treated mice showed an AHR decrease similar to that achieved using CPEE.

### CPEE Decreases MMP-9, ICAM-1 and VCAM-1 Expression and MMP-9 Activity in Lung Tissue of OVA-challenged Mice

To determine the possible protective mechanism underlying the activity of CP in airway inflammation, we investigated the expression of MMP-9 and adhesion molecules related to infiltration of inflammatory cells in lungs of OVA-challenged mice. By zymography, MMP-9 activity was increased in OVA-challenged mice compared with negative control mice (Fig. 7A). However, administration of CPEE significantly reduced MMP-9 activity in lung tissue of OVA-challenged mice. In addition, the reduction in MMP-9 activity is consistent with the results for expression of MMP-9 protein in lung tissue. While control OVA-challenged mice showed increased MMP-9 expression, this was dramatically decreased in CPEE-treated mice (Fig. 7B).

As shown in Figure 8, OVA-challenged mice showed a significant increase in expression of ICAM-1 and VCAM-1 in lung tissue compared with negative controls. However, in CPEE-treated mice, we observed a meaningful reduction in the expression of ICAM-1 and VCAM-1 compared with control OVA-challenged mice.

### CPEE Exhibits Protective Effect on Allergic Asthma via the Reduction in MMP-9 Activity in Lung Tissue

In additional experiment to clarify action mechanism of CPEE, CPEE treated mice showed the significant reduction in IL-4, IL-5, IL-13 and eotaxin compared with OVA-challenged mice (Table 1). MMPI-I treated mice significantly decreased Th2 cytokines and eotaxin compared with OVA-challenged mice, which similar to result of CPEE treated mice. Moreover, mice treated CPEE with MMPI-I lower Th2 cytokines and eotaxin than OVA-challenge. Although significant difference between CPEE plus MMPI-I treated mice and CPEE or MMPI-I treated mice, CPEE plus MMPI-I treated mice decreased compared with CPEE or MMPI-I treated mice.

In gelatin zymograph, CPEE or MMP-I treated mice significantly decreased MMP-9 activity compared with the OVA-challenged mice (Figure 9). CPEE plus MMPI-I treated mice also markedly decreased MMP-9 activity compared with OVA-challenged mice. Although MMP-9 activity in CPEE plus MMPI-I treated mice was lower than CPEE treated mice and MMP-9 treated mice, there was not significant.

### CPEE has no Effects on AST and ALT in a Murine Model of Allergic Asthma

As shown in Fig. 10, OVA-challenged mice showed marked increase in AST and ALT compared with the normal controls. Montelukast treated mice also exhibited the increase in AST and ALT same as those of OVA-challenged mice. In contrast, CPEE (100 and 200 mg/kg) treated mice showed the reduction in AST and ALT levels compared with OVA-challenged mice or montelukast treated mice. These results may be related with the protective effects of CPEE as described previously [11].

## Discussion

We evaluated the anti-inflammatory effects of CPEE in an OVA-induced murine asthma model and investigated its possible mechanism of action. OVA-challenged mice showed increases in the expression of MMP-9, ICAM-1 and VCAM-1 proteins in the lung tissue and the level of IL-4, IL-5, and eotaxin in the BALF. However, CPEE-treated OVA-challenged mice showed decreases in the numbers of eosinophils and macrophages in BALF and lung tissue, a reduction in IL-4, IL-5, IL-13 and eotaxin levels in BALF, total IgE, OVA-specific IgE and OVA-specific IgG1, and AHR compared with control OVA-challenged mice. Administration of CPEE also reduced the expression of MMP-9, ICAM-1 and VCAM-1 and MMP-9 activity in lung tissue.

Th2 cells play a key role in the initiation and progression of allergic asthma by induction of cytokine secretion. In particular, IL-4 and IL-5 lead to eosinophil-rich inflammation in lungs, and enhanced IgE production and mucus hypersecretion by epithelial goblet cells [23]. However, CPEE treatment of OVA-challenged mice significantly reduced level of IL-4, IL-5, IL-13 and eotaixn in BALF and total IgE, OVA-specific IgE and OVA-specific IgG1 in serum. In addition, CPEE induced the decreases in the number of inflammatory cells such as eosinophils, neutrophils, lymphocytes and macrophages. These results were confirmed by histological analysis, which showed that CPEE inhibited inflammatory cell infiltration and mucus hypersecretion. In addition, AHR is a crucial maker and a measure of the bronchial constriction commonly found in asthmatic condition. In this study, administration of CPEE exhibits the significant reduction in AHR, which may be caused by decreasing the levels of Th2 cytokines and IgE. These results suggest that CPEE has protective effects in allergic asthma by reducing in the levels of Th2 cytokines.

Leukocyte−endothelial adhesion molecules are important in the recruitment and infiltration of inflammatory cells into inflammatory lesions. Representative cell adhesion molecules include ICAM-1 and VCAM-1. According to previous studies, ICAM-1 and VCAM-1 are upregulated on the bronchial vascular endothelium after bronchial allergen challenge in patients with asthma [24], [25]. Additionally, it was reported that cytokines such as IL-4 and eotaxin activate the rapid expression of ICAM-1 and VCAM-1 [26]–[28]. In this study, control OVA-challenged mice showed increases in expression of ICAM-1 and VCAM-1 protein in lung tissue, as well as the increases in the levels of IL-4 and IL-5 of BALF. In contrast, CPEE-treated OVA challenged mice showed decreased expression of ICAM-1 and VCAM-1 in lung tissue accompanied by a reduction in the levels of IL-4 and IL-5 in BALF. These results suggest that in CPEE attenuates the infiltration of inflammatory cells by reducing the expression of ICAM-1 and VCAM-1 in OVA-induced allergic asthma.

MMP-9 mediates tissue remodeling in the airway by regulating the infiltration of inflammatory cells. Airway tissue remodeling is characterized by increased goblet cell hyperplasia and the production of MMP-9 [29], [30]. MMP-9 is modulated by a variety of cytokines [31]–[33] and is associated with tissue remodeling and infiltration of inflammatory cells into inflammatory sites [34]. Recently, it was reported that MMP-9 expression is increased in the bronchoalveolar lavage and lung tissue [29], [35] and overexpression of MMP-9 is upregulated in association with the accumulation of inflammatory cells in the airway in a murine asthma model [10]. In the same model, MMP inhibitiors reduced inflammatory cell infiltration though reduction in ICAM-1 and VCAM-1 expression [7]. A recent study revealed a relationship between the expression of adhesion molecules and MMP-9 in allergic airway inflammation [36]. These findings have suggested that the modulation of MMP-9 might be a potential therapeutic strategy for allergen-induced asthma. In this study, we evaluated the activity of MMP-9 using gelatin zymography and the expression of MMP-9 protein in lung tissue. CPEE-treated OVA challenged mice showed reduced activity and protein expression of MMP-9 in lung tissue compared with control OVA-challenged mice. These results were consistent with the observed levels of cytokines, and expression of ICAM-1 and VCAM-1 protein. These results showed in dose-dependent manner, which are in agreement with *in vivo* experiment using rats [11]. In additional study, results of MMP-9 activity in CPEE treated mice were consistent with first study. MMPI-I treated mice also were observed the reduction in MMP-9 activity, which similar to results of CPEE treated mice. In CPEE plus MMPI-I treated mice, MMP-9 activity lower than that of OVA-challenged mice, which reduced when compared with that of MMPI-I treated mice. These results were consistent with results of Th2 cytokines and eotaxin. These findings demonstrated that administration of CPEE attenuates airway inflammation, at least, via partial down-regulation of MMP-9, resulting in reduced expression of ICAM-1 and VCAM-1.

In conclusion, administration of CPEE in this murine asthma model significantly decreased the number of inflammatory cells, particularly eosinophils in BALF and lung tissue, and reduced IL-4 IL-5, IL-13 and eotaxin in BALF and total IgE, OVA-specific IgE and OVA-specific IgG1 levels and AHR in serum after OVA challenge. These findings indicate that CPEE may effectively inhibit the progression of the airway inflammation of allergic asthma. In addition, the anti-inflammatory effects of CPEE were partially mediated by down-regulation of MMP-9 resulting in reduction of ICAM-1 and VCAM-1 expression.
